# Correlation of EGFR expression, gene copy number and clinicopathological status in NSCLC

**DOI:** 10.1186/s13000-014-0165-0

**Published:** 2014-09-17

**Authors:** Rania Gaber, Iris Watermann, Christian Kugler, Nils Reinmuth, Rudolf M Huber, Philipp A Schnabel, Ekkehard Vollmer, Martin Reck, Torsten Goldmann

**Affiliations:** Clinical and Experimental Pathology, Research Center Borstel, Parkallee 3, 23845 Borstel, Germany; Pathology, Faculty of Medicine, Alexandria University, Chamblion street, el azareeta, Alexandria, Egypt; Center of Pulmonology and Thoracic Surgery, Lungenclinic Grosshansdorf, Wöhrendamm 80, 22927 Grosshansdorf, Germany; Airway Research Center North (ARCN), Member of the German Center for Lung Research, Borstel, Germany; Ludwig Maximilians University (LMU), Member of the German Center for Lung Research, Ziemssenstr. 1, 80336 München, Germany; Comprehensive Pneumology Center Munich, (CPC-M), Member of the German Center for Lung Research, München, Germany; Institute of Pathology, University Hospital Heidelberg, Im Neuenheimer Feld 224, 69120 Heidelberg, Germany; Translational Lung Research Center (TLRC), Member of the German Center for Lung Research, Heidelberg, Germany

**Keywords:** Non-small cell lung cancer (NSCLC), Epidermal Growth Factor Receptor (EGFR), Immunohistochemistry, Fluorescence in Situ Hybridization, Antibody clones

## Abstract

**Background:**

Epidermal Growth Factor Receptor (EGFR) targeting therapies are currently of great relevance for the treatment of lung cancer. For this reason, in addition to mutational analysis immunohistochemistry (IHC) of EGFR in lung cancer has been discussed for the decision making of according therapeutic strategies. The aim of this study was to obtain standardization of EGFR-expression methods for the selection of patients who might benefit of EGFR targeting therapies.

**Methods:**

As a starting point of a broad investigation, aimed at elucidating the expression of EGFR on different biological levels, four EGFR specific antibodies were analyzed concerning potential differences in expression levels by Immunohistochemistry (IHC) and correlated with fluorescence in situ hybridization (FISH) analysis and clinicopathological data. 206 tumor tissues were analyzed in a tissue microarray format employing immunohistochemistry with four different antibodies including Dako PharmDx kit (clone 2-18C9), clone 31G7, clone 2.1E1 and clone SP84 using three different scoring methods. Protein expression was compared to FISH utilizing two different probes.

**Results:**

EGFR protein expression determined by IHC with Dako PharmDx kit, clone 31G7 and clone 2.1E1 (p ≤ 0.05) correlated significantly with both FISH probes independently of the three scoring methods; best correlation is shown for 31G7 using the scoring method that defined EGFR positivity when ≥ 10% of the tumor cells show membranous staining of moderate and severe intensity (p = 0.001).

**Conclusion:**

Overall, our data show differences in EGFR expression determined by IHC, due to the applied antibody. Highest concordance with FISH is shown for antibody clone 31G7, evaluated with score B (p = 0.001). On this account, this antibody clone might by utilized for standard evaluation of EGFR expression by IHC.

**Virtual slides:**

The virtual slide(s) for this article can be found here: http://www.diagnosticpathology.diagnomx.eu/vs/13000_2014_165.

## Background

Lung cancer is the leading cause of death related to cancer in the world according to WHO data published in December 2013. Non-small cell lung cancer (NSCLC) accounts about 85% of all lung cancers [1]. Despite therapeutic advances, the overall 5-year survival is only 15% [2]. EGFR is a cell surface tyrosine kinase receptor abundantly expressed on all epithelial and stromal cells [3]. Expression of EGFR is deregulated in a variety of solid tumors and has been correlated with disease progression and poor survival [4]. In 34% to 84% of NSCLC patients, EGFR overexpression is also detectable; an increased expression of EGFR is proposed to be of prognostic and also of potential predictive relevance [5]. High EGFR gene copy numbers are found in almost 60% of the patients [6]. Based on its central role in cellular tumor growth, EGFR is intended as favored drug target for the development of specific anti-NSCLC treatments [7]. Plenty of EGFR specific therapeutics have been developed and tested in clinical trials; including specific antibodies such as cetuximab and necitumumab, as well as small molecule tyrosine kinase inhibitors (TKI) like erlotinib, afatinib, and gefitinib [8]. The identification of patients who might profit from these selective drugs is of tremendous interest. Although EGFR targeted therapies have been approved, there exists no general consensus concerning the evaluation of EGFR expression patterns in NSCLC. As shown in the FLEX-study (First Line Treatment for Patients with EGFR-expressing Advanced NSCLC), high EGFR H-scores can predict survival benefit for cetuximab plus first-line chemotherapy in patients with advanced NSCLC [9]. Due to the prognostic role of EGFR and the relevance of determination the EGFR expression status as well as the identification of EGFR mutations to select individual therapies for lung cancer patients, the evaluation of lung carcinomas require the optimal characterization of clinical sections in routine histopathology. Thus, it is of great relevance to determine the specific EGFR status to identify patients for appropriate therapies. With the ongoing progress in generation of EGFR-specific therapeutics, pathologists have to employ standardized protocols for defined antibodies used for immunohistochemical detection of EGFR expression as well as consistent scoring systems. So far, determination of EGFR status was performed by immunohistochemistry on paraffin-embedded tumor specimens to select patients suitable for EGFR-specific therapies. However, this method depends highly on the choice of the first antibody and the applied scoring method [10]. Since there are no data available concerning optimal selection of antibody used for diagnostic approaches, we compared four commercially available EGFR-specific antibodies and three different scoring systems concerning their disparities in immunohistochemical evaluation to obtain insight which variant comes off best for the determination of EGFR expression in NSCLC.

There are inconsistent data for the relationship between EGFR expression on protein level and response to EGFR specific therapies [11]. Nevertheless, an increased EGFR gene copy number has recently been proposed as predictor of anti EGFR targeted therapies in lung cancer patients [6]. The evaluation of EGFR gene status by FISH is delicate: EGFR gene variations in tumor cells are focal and low levels of EGFR amplification are difficult to visualize. As a start of an investigation, aimed to identify enlarged collectives of patients who might benefit from TKI treatment additionally to those, bearing activating mutations, we evaluated IHC-based methods to optimize the detection of EGFR expression on protein level using different fixation procedures. First, we analyzed immunohistochemistry and FISH in formalin-fixed, paraffin-embedded tissues (FFPE). FISH analysis was performed by application of two different probes to evaluate the EGFR gene status. Data were correlated with EGFR expression on protein level determined by IHC, in order to figure out the predictive value of EGFR expression on protein level and gene amplification status. Data of IHC and FISH analysis were correlated with clinicopathological data to find out, whether IHC could be the method of choice, probably coupled to FISH analysis. Thus, the objectives of these studies were first to investigate different antibodies and scoring systems in immunohistochemistry and the comparison of two different FISH probes. Second, to clarify if IHC correlates effectively with FISH-analysis. To evaluate the significance of EGFR determinations, tissues of 206 lung cancer patients were analyzed including their clinical data.

## Methods

### Patient data and tissues

For the construction of tissue microarray (TMA) blocks, a collection of 206 lung tumor surgical resection specimens with NSCLC were obtained after resection from the surgical department of LungenClinic Grosshansdorf (Table 1). The retrospective investigation included 100 cases of adenocarcinoma (ADC), 86 cases of squamous cell carcinoma (SCC), 12 cases of large cell carcinoma (LCC), 6 cases of carcinoid tumor and 2 cases of adenosquamous carcinoma. All tumor samples were histologically classified according to the International Association for the Study of Lung Cancer/American Thoracic Society/European Respiratory Society International Multidisciplinary classification of lung adenocarcinoma 2011 [12] and WHO guidelines 2010 [13]. Formalin fixed paraffin embedded blocks were collected from the Archive of Clinical & Experimental Pathology, Research Center Borstel, Germany.

**Table 1:** **Characteristics of 206 patients with non-small cell lung cancer**

**Category**	**Subcategory**	**Results (%)**
Age	≥ 65	128 (62.1)
	< 65	78 (37.9)
Gender	Male	132 (64)
	Female	74 (36)
*Smoking status	Current	78 (37.9)
	Former	30 (14.6)
	Never	12 (5.8)
*Asbestos contact	Present	17 (8)
	Absent	49 (24)
*COPD	Present	43 (21)
	Absent	5 (2)
Histologic type	ADC	100 (48.6)
	SCC	86 (41.7)
	LCC	12 (5.8)
	Other	8 (3.9)
ADC subtypes	Acinar predominant	38 (18.4)
	Solid predominant	25 (12.1)
	Papillary predominant	22 (10.8)
	Micropapillary predominant	10 (4.9)
	Invasive mucinous	3 (1.4)
	Lepidic	2 (1)
Grade	Well	5 (2.4)
	Moderate	82 (39.8)
	Poor	119 (57.8)
Tumor size	T1	39 (18.9)
	T2	115 (55.8)
	T3	32 (15.5)
	T4	20 (9.8)
Lymph node status	N0	99 (48.1)
	N1	49 (23.8)
	N2	43 (20.9)
	N3	15 (7.3)
Stage	I	68 (33)
	II	57 (27.7)
	III	73 (35.4)
	IV	8 (3.9)

*History of smoking, contact with asbestos and chronic obstructive pulmonary disease (COPD) were undetermined in the rest of the patients. Statistical analysis was done with available data.

### Ethics statement

This study was performed in compliance with the ethical committee of the University of Lübeck (reference number 12–220).

### Construction of tissue microarrays

For the construction of the TMAs, representative tumor punches (2 mm in diameter) were taken after characterization with Hematoxylin and Eosin staining (H&E) as previously described [14]. Two core biopsies from two different viable parts of each tumor specimen were transferred using the Beecher manual arrayer (Beecher instruments, Alpha, Metrix Biotech), in order to enhance representatives when analyzing the expression of EGFR. Paraffin embedded A549 cells were used as positive control.

### Immunohistochemistry

EGFR protein expression was assessed by immunohistochemistry on 2 μm deparaffinized TMA sections, using four EGFR specific antibodies: the Food and Drug Administration (FDA) approved Dako EGFR PharmDx kit (clone 2-18C9, mouse monoclonal, prediluted, DAKO, Corp., Glostrup, Denmark), Zymed antibody (clone 31G7, mouse monoclonal, 1:30, Zymed laboratories, San Francisco, CA), Zytomed antibody (clone 2.1E1, mouse monoclonal, 1:100, Zytomed Systems, Berlin, Germany) and antibody clone SP84 (rabbit monoclonal, 1:100, Spring Bioscience, CA). The first two clones are both recognizing the extracellular domain of EGFR and the mutant form of EGFR (EGFRvIII) by immunohistochemical staining [15] and Western Blot Analysis [16]. Antibody clone 2.1E1 does also recognize the extracellular part of the EGFR (Zytomed Information). In contrast, antibody clone SP84 is generated against a synthetic peptide corresponding to C-terminus of the EGFR protein.

Staining procedures were conducted according to manufacturer’s protocols. Antibodies were titrated for optimal sensitivity. Each TMA paraffin block was cut into multiple 2 μm thick sections, mounted on the positively charged slides and stained by H&E as well as with every of the EGFR specific antibodies. In each run of immunostaining, a separate negative control section was included where we omitted the primary antibody. For Dako PharmDx clone 2-18C9, a control slide was provided (Cell line CAMA-1 with expression level 0 and cell line HT-29 with expression level 2+) which was included in the IHC staining runs. For Dako PharmDx, 31G7 and 2.1E1, slides were deparaffinized, hydrated and antigen retrieval was performed with proteinase K. For SP84, antigen retrieval was done with 0.1 sodium citrate buffer, pH 6.0 (Merck KGaA, Darmstadt, Germany) microwaved for 4 minutes followed by 30 minutes cooling at RT. Blocking of endogenous peroxidase was achieved by immersing the sections in 3% H_2_O_2_ for 10 minutes at RT (Dako PharmDx and Merck KGaA, Darmstadt, Germany), then washed in TRIS buffer (10× Dako PharmDx kit wash buffer and 10× Zytomed biosystems wash buffer) for 2 minutes. Subsequently, sections were incubated with the different clones of anti-EGFR antibody (Dako PharmDx negative control reagent and Zytomed biosystems antibody diluent) for one hour in humidified chambers at RT. Sections, stained with 31G7 and 2.1E1 were incubated at RT with Post Block reagent for 15 minutes before HRP polymer was added for 20 minutes (Zytomed Systems). For SP84 HRP was incubated for 20 minutes and for Dako PharmDx (Zytomed systems and Dako labeled polymer HRP) for 30 minutes respectively. Sections were washed in TRIS buffer triply for two minutes after incubation with each reagent. DAB substrate kit (DAB chromogen and DAB substrate) was used for 15 minutes to visualize antibody binding. At the end, counterstaining of the sections was performed in Meyer’s hematoxylin. Finally, sections were mounted with Pertex (Medite GmbH, Burgdorf, Germany).

### EGFR scoring methodology

Specimens were evaluated by light microscopy (Nikon Eclipse 50i) using low (×100) and high (×200 or ×400) magnification.

The EGFR expression by IHC was scored using three different scoring methods:

(A) H-score: as applied in the retrospective FLEX study [17] is the product of the percentage of cancer cells positive for EGFR protein on the cell surface multiplied by the overall intensity of staining (ranging from 0 to 3+), producing a number from 0 to 300 [9,18].
(B) EGFR expression is defined as positive, if ≥10% of the tumor cells, using ×10 and ×20 magnification, show membranous staining of only 2+ and 3+ [6,19,20].
(C) EGFR expression is considered as positive, if ≥10% of the tumor cells show membranous staining of any intensity using ×10 and ×20 magnification assessed by Dako EGFR PharmDx data sheet.

Assessment of EGFR IHC was done for each single core by two independent observers and the mean of the two cores was used as a result for the EGFR expression of each case. In addition, single sections of the original FFPE blocks were stained with the four EGFR-specific antibodies of: 1) All cases of LCC; carcinoid and adenosquamous carcinoma (tumors with low frequency), 2) 12 cases which lost one of the two cores in the arrays during IHC, were complemented by staining of whole cut sections.

### Fluorescence in Situ Hybridization

Two different FISH probes of EGFR/Centromere of chromosome 7 (CEN7, CEP7) were used in the study: Dako Cytomation FISH probe mix (DAKO; Denmark, A/S) and ZytoLight SPEC EGFR/CEN 7 dual color probe (Zytomed system, Berlin, Germany). The FISH assay and analysis of each TMA was done with both probes (n = 412).

Dual color (FISH) was performed on 2 μm thick-sections. Before hybridization, sections were deparaffinized, dehydrated and immersed in citrate buffer (Merck KGaA, Darmstadt, Germany) pH 6 at 98°C for 15 minutes, followed by 2 minutes in distilled water twice. The sections were air dried and pretreated with pepsin for 5 minutes before denatured for 10 minutes at 75°C. After overnight hybridization at 37°C, slides were washed and counterstained with 1.5 μg/ml 4′,6′-diamidino-2-phenylindole (DAPI) mounting medium (Vectashield, Vector laboratories, Burlingame, CA) and coverslips were fixed with nail polish.

Analysis of FISH signals was performed on an epifluorescence microscope Nikon Eclipse 80i H550L (Nikon) with interference filters (AHF Analysentechnik AG, Tübingen, Germany).

At least 50 non-overlapped interphase nuclei of average size were scored per core. The selection of the nuclei was done using the DAPI filter under high magnification (×600). For each probe, the number of the EGFR and the chromosome 7 centromere per nuclei were visualized and scored using the green (FITC) and red (ET Rhod) filters separately as well as the double red and green filter. The red filter was used to visualize the EGFR sequence of Dakocytomation FISH probe mix and the chromosome 7 centromere of Zytolight SPEC EGFR/CEN7 dual color probe. While the green filter was used to visualize the EGFR sequence of Zytolight SPEC EGFR/CEN7 dual color probe and the chromosome 7 centromere of Dako cytomation FISH probe mix.

EGFR gene status results were grouped according to the Colorado scoring system, classified into six main categories [21]. 1) disomy: ≤ 2 copies in > 90% of the cells, 2) low trisomy: ≤ 3 copies in ≥ 40% of cells, 3 copies in 10% – 40% of the cells, ≥ 4 copies in < 10% of cells, 3) high trisomy: ≤ 3 copies in ⊔ 40% of cells, 3 copies in ≥ 40% of cells, ≥ 4 copies in < 10% of cells, 4) low polysomy: ≥ 4 copies in 10% – 40% of cells, 5) high polysomy: ≥ 4 copies in ⊔ 40% of cells, 6) gene amplification: specimens with EGFR gene amplification, defined as: (a): EGFR gene to CEP 7 ratio ≥ 2, (b): small gene clusters (4 – 10 copies) or innumerable tight gene cluster in > 10% the tumor cells independent of the EGFR to CEP 7 ratio, (c): larger and brighter EGFR signals than CEP 7 signals in > 10% of the tumor cells, while EGFR signals are smaller than the CEP 7 signals in the adjacent stromal and reactive cells independent of the EGFR to CEP 7 ratio, (d): > 5 copies of the EGFR signals in > 10% of tumor cells independent of the EGFR to CEP 7 ratio. The gene amplification was classified into low and high levels according to gene to chromosome ratio ranged between 2.1 and 3 for low amplification and more than 3 for high amplification [22–24]. Finally, patients were grouped into EGFR FISH-negative (disomy, low trisomy, high trisomy, low polysomy) and EGFR FISH-positive (high polysomy, low amplification, high amplification). The assessment of the gene copy number was done for each single core for each case and the core with the highest copy number was used as a result of the FISH assay.

As for the IHC, additional single sections of the original FFPE blocks of representative cases of the tumors with low frequency and the cases which lost one of the two cores as assessed by H&E were cut. The tumor area was selected and marked and one of the FISH probes of EGFR/CEN 7 was applied to this area for the analysis and the comparison between the whole cut section and the cores included in the arrays.

### Statistical analysis

The statistical analysis was performed using SPSS version 20 (SPSS Inc., Chicago, IL). Associations between the different variables were done using chi-square test and Mann-Mann–Whitney U test. The tests were double sided. Differences were considered statistically significant for p values <0.05.

## Results

### Patient characteristics

From the 206 patients, 100 tumors (48.6%) were classified as ADC, 86 tumors (41.7%) were grouped as SCC, 12 tumors (5.8%) as LCC, and 8 tumors (3.9%) as other tumors (4 typical carcinoids, 2 atypical carcinoids and 2 adenosquamous carcinoma). Basal characteristics of the patients and clinicopathological status are summarized in Table 1. According to WHO 2010, pathological TNM staging was IA in 22 (10.8%), IIA in 38 (18.4%), IIIA in 53 (25.7%), IB in 46 (22.3%), IIB in 19 (9.2%), IIIB in 20 (9.7%) and IV in 8 (3.9%) patients. Smoking history was available for 120 cases and classified as current, former and never smokers [25]. Contact with asbestos was inquired by asking the patients.

The age of the patients showed statistical significant differences for the stage and the grades of tumors (p = 0.034 and p = 0.009). From the patients ≥65 years old, 49 (38.3%) had tumors stage I. 65 (54.6%) had poorly differentiated tumors. Patients <65 years old, 35 (44.9%) had tumors stage III and 54 (69.2%) had poorly differentiated tumors. The gender of the patients and different grades of tumors displayed a significant difference within histological subtypes: 68 (51.5%) of the male patients had SCC and 48 (64.9%) of female patients had ADC (p < 0.001). For the grades of the carcinomas: 66 tumors (55.5%) and 39 tumors (32.8%) of poorly differentiated carcinomas were ADC and SCC respectively, while 45 tumors (54.9%) of moderately differentiated cases were SCC (p < 0.001).

Significant differences were found between ADC subtypes and grades: 24 (36.4%) tumors of poorly differentiated ADC were grouped as acinar predominant. 23 tumors (34.8%) were classified as solid predominant with mucin production. 8 cases (12.1%) belonged to papillary predominant, 8 tumors (12.1%) were of micropapillary predominant, 2 tumors (3%) of lepidic and 1 case (1.5%) of invasive mucinous. Smoking behavior was significantly associated with the histological subtypes and the grades. 36 patients (46.2%) of the current smokers, and 18 patients (60%) of former smokers had SCC; while 8 patients (66.7%) of never smokers had ADC (p = 0.023). 53 SCC (67.9%) were of current smokers. 16 patients (53.3%) of the former smokers had poorly differentiated tumors and 6 patients (50%) of the never smokers had moderately differentiated tumors (p = 0.001). Contact with asbestos was significantly associated with stage. 10 (55.6%) cases who had contact with asbestos were stage I and 23 (45.1%) of those who did not have asbestos contact were stage III (p = 0.039). Correlations among clinicopathological parameters were not found.

### Comparison of different EGFR-specific antibodies and three scoring methods

All of the full sections taken for tumors with a rare incidence and 12 cases which lost one of their two cores showed the same results as their respective cores in the arrays. Results of the statistical association of the four different EGFR specific antibodies and the three different scoring methods are shown in Table 2.

**Table 2:** **EGFR expression evaluated with Dako PharmDx, 31G7, 2.1E1 and SP84 using three different scoring methods; correlation between the four antibodies**

		**31G7**		**2.1E1**		**SP84**	
		**Positive**	**Negative**	**Positive**	**Negative**	**Positive**	**Negative**
**Score A**							
**DAKO**	**Positive**	116(56.3%)	13(6.3%)*	129(62.6%)	0(0%)*	103(50%)	26(12.6%)*
	**Negative total**	21(10.2%) 137(66.5%)	56(27.2%) 69(33.5%)	48(23.3%) 177(85.9%)	29(14.1%) 29(14.1%)	9(4.4%) 112(54.4%)	68(33%) 94(45.6%)
**31G7**	**Positive**			137(66.5%)	0(0%)*	101(49%)	36(17.5%)*
	**Negative total**			40(19.4%) 177(85.9%)	29(14.1%) 29(14.1%)	11(5.3%) 112(54.3%)	58(28.2%) 94(45.7%)
**2.1E1**	**Positive**					112(54.3%)	65(31.6%)*
	**Negative total**					0(0%) 112(54.3%)	29(14.1%) 94(45.7%)
**Score B**							
**DAKO**	**Positive**	170(82.5%)	6(2.9%)*	175(85%)	1(0.4%)*	154(74.7%)	22(10.7%)*
	**Negative total**	6(2.9%) 176(85.4%)	24(11.7%) 30(14.6%)	13(6.3%) 188(94.3%)	17(8.3%) 18(8.7%)	6(2.9%) 160(77.6%)	24(11.7%) 46(22.4%)
**31G7**	**Positive**			175(85%)	1(0.4%)*	157(76.2%)	19(9.2%) *
	**Negative total**			13(6.3%) 188(91.3%)	17(8.3%) 18(8.7%)	3(1.5%) 160(77.7%)	27(13.1%) 46(22.3%)
**2.1E1**	**Positive**					158(76.6%)	30(14.6%)*
	**Negative total**					2(1%) 160(77.6%)	16(7.8%) 46(22.4%)
**Score C**							
**DAKO**	**Positive**	183(88.8%)	1(0.5%)*	183(88.8%)	1(0.5%)*	177(85.9%)	7(3.4%)*
	**Negative total**	2(1%) 185(89.8%)	20(9.7%) 21(10.2%)	7(3.4%) 190(92.2%)	15(7.3%) 16(7.8%)	4(2.0%) 181(87.9%)	18(8.7%) 25(12.1%)
**31G7**	**Positive**			185(89.8%)	0(0%)*	178(86.4%)	7(3.4%)*
	**Negative total**			5(2.4%) 190(92.2%)	16(7.8%) 16(7.8%)	3(1.5%) 181(87.9%)	18(8.7%) 25(12.1%)
**2.1E1**	**Positive**					179(86.9%)	11(5.3%)*
	**Negative total**					2(1%) 181(87.9%)	14(6.8%) 25(12.1%)

*p < 0.001.

**Figure 1:**
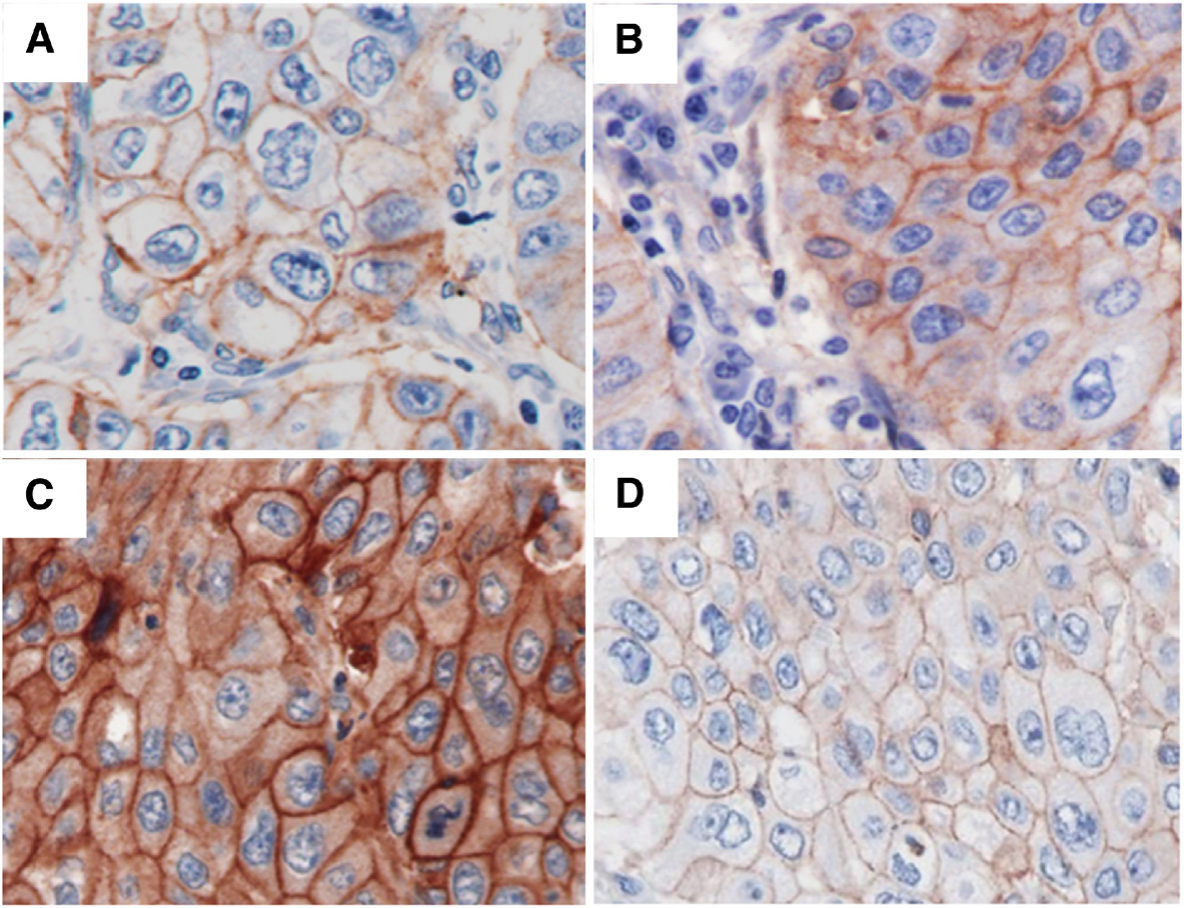
**Immunohistochemical EGFR staining with four different antibodies showing differences in levels of EGFR expression in the same specimen of a squamous cell carcinoma (SSC) (original magnification × 400). (A)** Staining intensity with Dako PharmDx 2+, **(B)** Staining intensity with 31G7 2+, **(C)** Staining intensity with 2.1E1 3+, **(D)** Staining intensity with SP84 1+.

**Figure 2:**
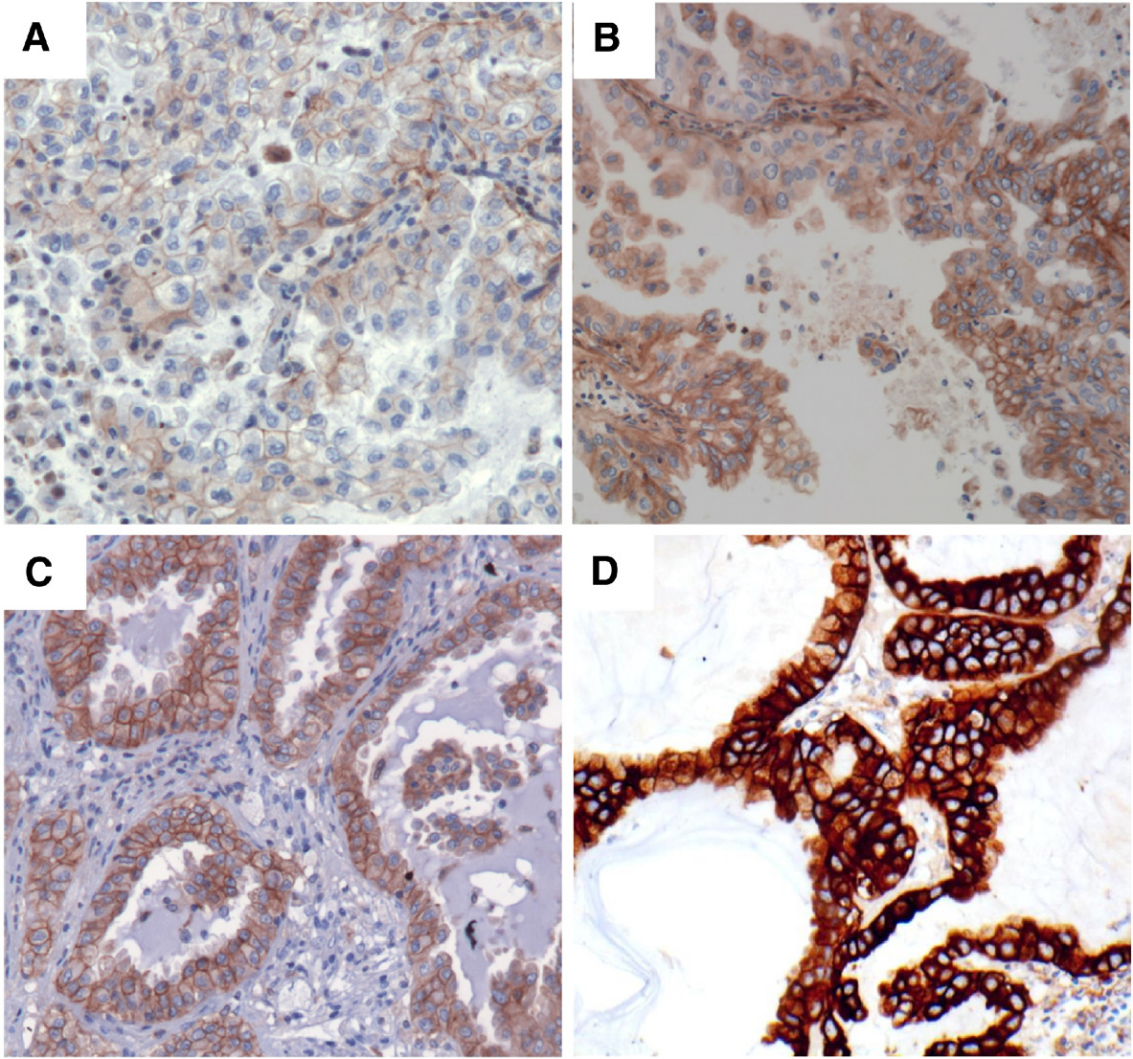
**Different cases of adenocarcinomas (ADC) showing different results using scores A, B, C (original magnification × 400). (A)** Positive with score C, negative in A and B (intensity 1 in 100% of tumor cells H-score ⊔ 200). **(B)** Positive with score B and C and negative in score A (Tumor cells show different intensities: 2 in 40%, 1 in 40% and 0 in 20% H-score ⊔ 200). **(C)** and **(D)** Positive in all scores: **C)** intensity 2 in 100% of tumors cells H-score = 200, **D)** intensity 3 in 100% of tumor cells H-score = 300).

Intensity of EGFR immunostainings, performed with the four different antibody clones varied both within one tumor sample as well as in different tumor specimens (Figure 1). Analyzing the expression patterns of EGFR using 3 scoring methods maintained different results for EGFR positivity (Figure 2). Of 206 patients, analyzed with Dako pharmDx and evaluated with scoring method (A), 129 tumors samples (62.6%) were allocated as positive. Analyzing EGFR immunostaining of Dako pharmDx with scoring method (B) determined 176 patients (85.4%) as positive and scoring method (C) showed positive EGFR staining in 184 samples (89.3%).

The outcome of applying, 31G7 using scoring method (A) yielded 137 EGFR positive tumors (66.5%). Whereas with scoring method (B), 176 tumors (85.4%) and for scoring method (C), 185 samples (89.9%) were defined as EGFR positive.

**Figure 3:**
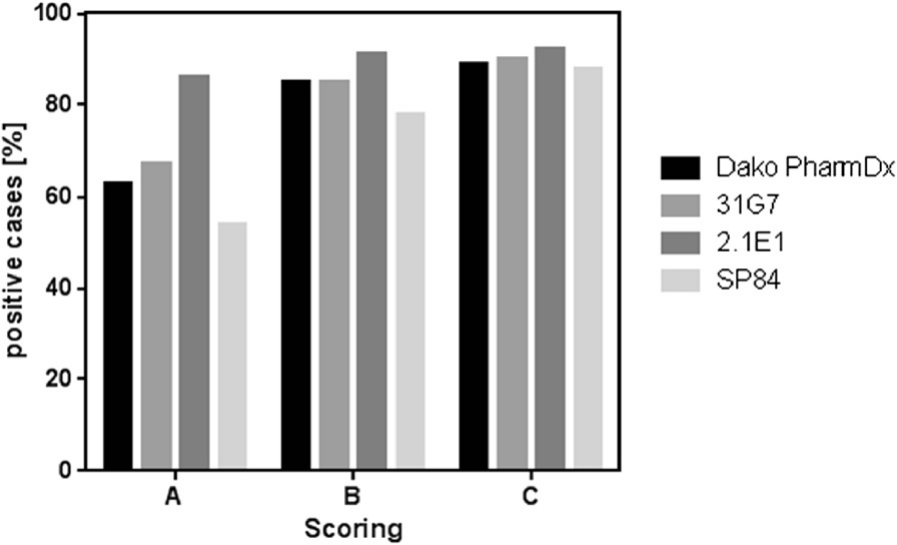
**Assessment of Dako PharmDx, 31G7, 2.1E1 and SP84 using three different scoring methods.**

Antibody 2.1E1 was the most sensitive antibody: we obtained highest numbers of EGFR-positive tumor samples using this clone in immunostainings for all of the three scoring methods. 31G7 and Dako pharmDx show similar staining intensities independent of the scoring methods, whereas SP84 showed the lowest sensitivity. Decreasing the cut off values, lead for all antibody clones to increasing numbers of EGFR positive immunostainings (Figure 3).

The degree of agreement determined with score (A) between Dako PharmDx compared with 31G7, 2.1E1 and SP84 was 83.5%, 76.7% and 83% respectively. Correlation between 31G7 and 2.1E1 and between 31G7 and SP84 amounts to 80.6% and 77.2% respectively, and between 2.1E1 and SP84, to 68.4%. For Score (B), the correlation between Dako PharmDx and 31G7 was 94.2%, for Dako PharmDx and 2.1E1 it was 93.3% and between Dako PharmDx and SP84 it was 86.4%. The agreement between 31G7 and 2.1E1 and 31G7 and SP84 was 93.3% and 89.3% respectively, between 2.1E1 and SP84 it constituted 84.4%. Scoring with method (C) showed a degree of agreement between Dako PharmDx, 31G7, 2.1E1 and SP84 of 98.5%, 96.1% and 94.6% respectively. Between 31G7 and 2.1E1 and SP84 it was determined as 97.6% and 95.1%, respectively and between 2.1E1 and SP84 it was 93.7% (Table 2).

All antibody clones investigated showed different degrees of agreement concerning the EGFR expression when analyzed by the three different scoring methods. For the Dako PharmDx antibody clone, the correlation between scoring method (A) and (B) was 73.3%, between (A) and (C) it was 70.1% and between (B) and (C) 95.7%. Antibody clone 31G7 depicted the degree of agreement between scoring method (A) and (B) of 77.8%, between (A) and (C) of 74.1% and between (B) and (C) of 95.1%. For antibody clone 2.1E1 correlation between scoring method (A) and (B) accounted to 94.1%, between (A) and (C) to 93.2%, and to 98.9% between scoring method (B) and (C). Antibody clone SP84 showed the degree of agreement between scoring method (A) and (B) of 70%, between (A) and (C) of 61.9% and between scoring method (B) and (C) of 88.4% (Table 2).

### Relationship between EGFR protein expression and clinicopathological data

The results for the four EGFR specific antibodies obtained by immunostaining and analyzed by three different scoring methods varied within the histological types of NSCLC.

The four antibody clones showed diverse intensities of staining and therefore different EGFR expression patterns within the same specimen leading to different IHC results (Figure 1).

Dependent of the scoring method, performed, assessment of EGFR expression varies. Different ADC tumor samples show varying results in EGFR expression according to the different scoring systems (A), (B) and (C) (Figure 2).

EGFR expression evaluated with scoring method (A) showed for all of the four antibodies fewer numbers of EGFR positive tumor samples for all of the four antibodies in SCC and ADC than evaluated with method (B) and (C). Data are shown in Table 2. Scoring with method (C) features more EGFR-positive tumor samples in ADC than in SCC. EGFR protein expression evaluated with 31G7, scored with method (B) and 2.1E1 scored with method (A) showed a significant association with tumor differentiation (p = 0.041 and p = 0.029).

An association between EGFR expression and tumor grade was shown when evaluation was performed with 31G7 using scoring method (B) (p = 0.02) and 2.1E1 employing scoring method (A) (p = 0.009) and (C) (p = 0.047). Smoking behavior correlates with EGFR expression when evaluated by IHC with antibody SP84 using scoring method (C) (p = 0.014).

EGFR protein expression determined with the four antibodies was not associated with age, sex, tumor stage and tumor or lymph node status.

### EGFR Fluorescence in Situ Hybridization

**Figure 4:**
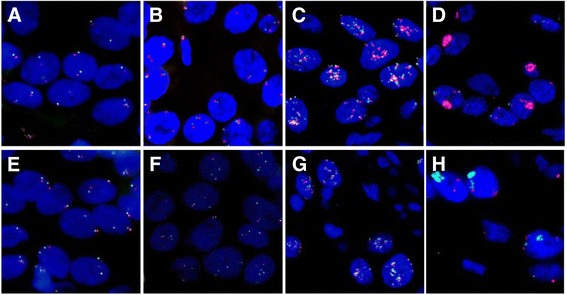
**FISH analysis with two different EGFR-specific FISH probes.** **A**, **B**, **C**, **D**: Dako Cytomation FISH probe mix (EGFR: red, CEN7: green), **E**, **F**, **G**, **H**: ZytoLight SPEC EGFR/CEN7 dual probe (EGFR: green, CEN7: red), (magnification × 630) **A**, **E**: balanced disomy, **B**, **F**: balanced trisomy, **C**, **G**: low amplification, **D**, **H**: high amplification.

FISH analysis for EGFR gene copy numbers showed exactly the same results for both probes (Dako Cytomation FISH probe mix and ZytoLight SPEC) (Figure 4). Of 206 tumor samples, 84 tumors (40.8%) showed disomy, 36 tumors (17.5%) low trisomy, 22 tumor samples (10.7%) high trisomy, 22 tumor samples (10.7%) low polysomy, 28 patients (13.6%) high polysomy, 5 tumors (2.4%) low amplification, and 9 patients (4.4%) high amplification. Altogether, positive FISH results including high amplification, low amplification and high polysomy were demonstrated in 42 cases (20.4%).

### Association between EGFR copy number and clinicopathological data

FISH positivity was more frequently in ADC than in SCC and LCC (23 (11.2%) vs 17 (8.3%) vs 2 (1%). Differences did not reach statistical significance (p = 0.567) (Table 3). Thus, the existence of amplification did not correlate with the histological subtypes.

**Table 3:** ᅟ

		**Total N (%)**	**Dako pharmDx**			**31G7**			**2.1E1**			**SP84**			**FISH**
			**A**	**B**	**C**	**A**	**B**	**C**	**A**	**B**	**C**	**A**	**B**	**C**	**+**
**Age**	≥65	128(62)	84(40.8)	112(54.4)	118(57.3)	90(43.7)	112(54.4)	117(56.8)	112(54.4)	119(57.8)	119(57.5)	72(35)	98(47.6)	113(54.9)	26(12.6)
	<65	78(38)	45(21.8)	64(31.1)	66(32)	47(22.8)	64(31.1)	68(33)	65(31.6)	69(33.5)	71(34.5)	40(19.4)	62(30.1)	68(33)	16(7.8)
		***P value***	0.299	0.312	0.105	0.171	0.312	0.350	0.416	0.312	0.789	0.564	0.731	0.829	1
**Sex**	Male	132(64)	81(39.3)	115(55.8)	121(58.7)	85(41.3)	116(56.3)	123(59.7)	115(55.8)	124(60.2)	125(60.7)	75(36.4)	109(52.9)	120(58.3)	24(11.7)
	Female	74(36)	48(23.3)	61(29.6)	63(30.6)	52(25.2)	60(29.1)	62(30.1)	62(30.1)	64(31.1)	65(31.6)	37(18)	51(24.8)	61(29.6)	18(8.7)
		***P value***	0.655	0.412	0.163	0.443	0.218	0.052	0.535	0.077	0.103	0.383	0.036	0.080	0.368
**Histology**	ADC	100(48.6)	53(25.7)	82(39.8)	88(42.7)	58(28.2)	81(39.3)	89(43.2)	83(40.3)	90(43.7)	91(44.2)	42(20.4)	67(32.5)	85(41.3)	23(11.2)
	SCC	86(41.7)	69(33.5)	82(39.8)	84(40.8)	70(34)	83(40.3)	84(40.8)	83(40.3)	84(40.8)	85(41.3)	65(31.6)	82(39.8)	84(40.8)	17(8.3)
	LCC	12(5.8)	6(2.9)	9(4.4)	9(4.4)	7(3.4)	9(4.4)	9(4.4)	8(3.9)	9(4.4)	9(4.4)	5(2.4)	9(4.4)	9(4.4)	2(1)
	Other	8(3.9)	1(0.5)	3(1.5)	3(1.5)	2(1)	3(1.5)	3(1.5)	3(1.5)	5(2.4)	5(2.4)	0(0)	2(1)	3(1.5)	0(0)
		***P value***	0.000	0.000	0.000	0.000	0.000	0.000	0.000	0.001	0.000	0.000	0.000	0.000	0.567
**ADC subtypes**	Acinar	38(18.4)	21(21)	30(30)	33(33)	24(24)	32(32)	33(33)	31(31)	33(33)	33(33)	16(16)	26(26)	34(34)	11(11)
	Solid	25(12.1)	11(11)	19(19)	21(21)	12(12)	19(19)	22(22)	19(19)	22(22)	22(22)	8(8)	16(16)	21(21)	2(2)
	Papillary	22(10.8)	15(15)	21(21)	22(22)	16(16)	21(21)	22(22)	22(22)	22(22)	22(22)	12(12)	17(17)	20(20)	7(7)
	Micro papillary	10(4.9)	4(4)	8(8)	8(8)	4(4)	6(6)	8(8)	8(8)	9(9)	9(9)	4(4)	5(5)	6(6)	1(1)
	Invasive mucinous	3(1.4)	1(19	2(2)	2(2)	1(1)	2(2)	2(2)	2(2)	2(2)	2(2)	1(1)	2(2)	2(2)	2(2)
	Lepidic	2(1)	1(1)	2(2)	2(2)	1(1)	1(1)	2(2)	1(1)	2(2)	3(3)	1(1)	1(1)	2(2)	0(0)
		***P value***	0.516	0.348	0.181	0.302	0.041	0.201	0.029	0.258	0.499	0.751	0.438	0.189	0.029
**Grade**	Well	5(2.4)	2(1)	3(1.5)	3(1.5)	2(1)	2(1)	3(1.5)	2(1)	3(1.5)	3(1.5)	2(1)	2(1)	3(1.5)	1(0.5)
	Moderate	82(39.8)	55(26.7)	71(34.5)	73(35.4)	60(29.1)	72(35)	74(35.9)	74(35.9)	76(36.9)	77(37.4)	46(22.3)	67(32.5)	72(35)	17(8.3)
	poor	119(57.8)	72(35)	102(49.5)	108(52.4)	75(36.4)	102(49.5)	108(52.4)	101(49)	109(52.9)	110(53.4)	64(31.1)	91(44.2)	106(51.5)	24(11.7)
		***P value***	0.371	0.268	0.107	0.148	0.020	0.126	0.009	0.62	0.047	0.789	0.075	0.155	1
**Tumor size**	T1	39(18.9)	24(11.7)	30(14.6)	31(15)	25(12.1)	31(15)	32(15.5)	30(14.6)	33(16)	34(16.5)	20(9.7)	28(13.6)	31(15)	5(2.4)
	T2	115(55.9)	71(34.5)	99(48.1)	105(51)	78(37.9)	99(48.1)	104(50.5)	100(48.5)	104(50.5)	105(51)	61(29.6)	90(43.7)	102(49.5)	24(11.7)
	T3	32(15.5)	21(10.2)	30(14.6)	31(15)	20(9.7)	29(14.1)	31(15)	28(13.6)	31(15)	31(15)	17(8.3)	24(11.7)	29(14.1)	7(3.4)
	T4	20(9.8)	13(6.3)	17(8.3)	17(8.3)	14(6.8)	17(8.3)	18(8.7)	19(9.2)	20(9.7)	20(9.7)	14(6.8)	18(8.7)	19(9.2)	6(2.9)
		***P value***	0.982	0.271	0.080	0.919	0.615	0.234	0.293	0.181	0.309	0.539	0.444	0.362	0.449
**Lymph node**	N0	99(48)	66(32)	82(39.8)	86(41.7)	66(32)	82(39.8)	86(41.7)	82(39.8)	88(42.7)	88(42.7)	54(26.2)	75(36.4)	84(40.8)	14(6.8)
	N1	49(23.8)	30(14.6)	43(20.9)	46(22.3)	31(15)	43(20.9)	46(22.3)	44(21.4)	46(22.3)	46(22.3)	26(12.6)	39(18.9)	44(21.4)	13(6.3)
	N2	43(20.9)	24(11.7)	38(18.4)	38(18.4)	27(13.1)	37(18)	39(18.9)	37(18)	40(19.4)	41(19.9)	25(12.1)	33(16)	39(18.9)	12(5.8)
	N3	15(7.3)	9(4.4)	13(6.3)	14(6.8)	13(6.3)	14(6.8)	14(6.8)	14(6.8)	14(6.8)	15(7.3)	7(3.4)	13(6.3)	14(6.8)	3(1.5)
		***P value***	0.643	0.835	0.667	0.358	0.765	0.645	0.652	0.833	0.460	0.892	0.843	0.741	0.151
**Stage**	I	68(33)	45(21.8)	57(27.7)	60(29.1)	47(22.8)	59(28.6)	60(29.1)	59(28.6)	62(30.1)	62(30.1)	36(17.5)	53(25.7)	58(28.2)	8(3.9)
	II	57(27.7)	32(15.5)	46(22.3)	50(24.3)	34(16.5)	44(21.4)	49(23.8)	45(21.8)	49(23.8)	49(23.8)	27(13.1)	40(19.4)	47(22.8)	13(6.3)
	III	73(35.4)	45(21.8)	66(32)	67(32.5)	50(24.3)	66(32)	68(33)	65(31.6)	69(33.5)	71(34.5)	44(21.4)	60(29.1)	68(33)	18(8.7)
	IV	8(3.9)	7(3.4)	7(3.4)	7(3.4)	6(2.9)	7(3.4)	8(3.9)	8(3.9)	8(3.9)	8(3.9)	5(2.4)	7(3.4)	8(3.9)	3(1.5)
		***P value***	0.337	0.406	0.771	0.635	0.192	0.493	0.317	0.380	0.093	0.494	0.403	0.194	0.095
**Smoking behavior**	Current	78(56.1)	52(43.3)	71(59.2)	72(60)	55(45.8)	70(58.3)	72(60)	71(59.2)	72(60)	72(60)	44(36.7)	67(55.8)	72(60)	16(13.3)
	Former	30(21.6)	22(18.3)	27(22.5)	28(23.3)	23(19.2)	27(22.5)	29(24.2)	27(22.5)	29(24.2)	29(24.2)	19(15.8)	26(21.7)	29(24.2)	4(3.3)
	Never	12(8.6)	6(5)	9(7.5)	9(7.5)	6(5)	9(7.5)	9(7.5)	9(7.5)	9(7.5)	10(8.3)	5(4.2)	7(5.8)	8(6.7)	2(1.7)
		***P value***	0.342	0.254	0.150	0.254	0.373	0.078	0.254	0.078	0.368	0.494	0.055	0.014	0.729
**Asbestos contact**	Present	18(20.5)	14(20.3%)	17(24.6)	17(24.6)	14(20.3)	17(24.6%)	17(24.6)	16(23.2)	17(24.6%)	17(24.6)	12(17.4)	16(23.2%)	18(26.1)	3(4.3)
	Absent	51(58)	37(53.6%)	45(65.2)	45(65.2)	38(55.1)	44(63.8%)	46(66.7)	44(63.8)	46(66.7%)	46(66.7)	28(40.5)	40(58%)	46(66.7)	14(20.3)
		***P value***	0.763	0.667	0.667	1.000	0.441	0.681	1.000	0.681	0.681	0.421	0.489	0.316	0.528

But, for ADC an association between FISH positivity and subclassification was found. EGFR FISH positivity was evaluated for 11 (5.4%) tumors that were of acinar predominant subtype, 2 (1%) of solid predominant, 7 (3.4%) of papillary predominant, 1 (0.5%) of micropapillary predominant and 2 (1%) of lepidic subtype (p = 0.029) (Table 4).

**Table 4:** **Association between ADC subtypes and FISH patterns**

	**D**	**LT**	**HT**	**LP**	**HP**	**LA**	**HA**
Acinar	15(7.3%)	6(2.9%)	1(1%)	5(2.4%)	10(4.9%)	0(0%)	1(0.5%)
Solid	6(2.9%)	8(3.9%)	5(2.4%)	4(1.9%)	2(1%)	0(0%)	0(0%)
Papillary	6(2.9%)	2(1%)	2(1%)	5(2.4%)	5(2.4%)	1(0.5%)	1(0.5%)
Micropapillary	7(3.4%)	0(0%)	2(1%)	0(0%)	1(0.5%)	0(0%)	0(0%)
Invasivemucinous	2(1%)	0(0%)	0(0%)	1(0.5%)	0(0%)	0(0%)	0(0%)
Lepidic	0(0%)	0(0%)	0(0%)	0(0%)	1(0.5%)	1(0.5%)	0(0%)

The distribution of FISH patterns was not associated with age, sex, smoking, contact of asbestos or COPD, grade, stage, size of lymph nodes or tumor size (Table 3).

### Association between EGFR protein expression and EGFR copy number

There was a significant (Fisher’s exact test) association between IHC positivity and EGFR gene copy number per cell for all of the four investigated antibody clones independent of the applied scoring method, except for SP84 analyzed with score A and B (Table 5). Dako PharmDx, 31G7 and 2.1E1, evaluated by three different scoring methods revealed significant correlation to FISH analysis. Immunostainings with 31G7, evaluated with scoring method B showed the highest concordance with FISH analysis (Table 5).

**Table 5:** **Association between IHC using three different scoring methods and FISH analysis**

**EGFR IHC**			**FISH**		**Total**	**P value**
			**Positive**	**Negative**		
Dako PharmDx	Score(A)	Positive	34(16.5%)	95(46.1%)	129(62.6%) 77(37.4%)	0.007
		Negative	8(3.9%)	69(33.5%)		
	Score(B)	Positive	41(19.9%)	135(65.5%)	176(85.4%) 30(14.6%)	0.012
		Negative	1(0.5%)	29(14.1%)		
	Score(C)	Positive	42(20.4%)	142(68.9%)	184(89.3%) 22(10.7%)	0.009
		Negative	0(0%)	22(10.7%)		
31G7	Score(A)	Positive	34(16.5%)	61(29.6%)	95(46.1%) 111(53.9%)	0.028
		Negative	8(3.9%)	103(50%)		
	Score(B)	Positive	42(20.4%)	134(65%)	176(85.4%) 30(14.6%)	0.001
		Negative	0(0%)	30(14.6%)		
	Score(C)	Positive	42(20.4%)	143(69.4%)	185(89.8%) 21(10.2%)	0.009
		Negative	0(0%)	21(10.2%)		
2.1E1	Score(A)	Positive	41(19.9%)	136(66%)	177(85.9%) 29(14.1%)	0.012
		Negative	1(0.5%)	28(13.6%)		
	Score(B)	Positive	42(20.4%)	146(70.9%)	188(91.3%) 18(8.7%)	0.027
		Negative	0(0%)	18(8.7%)		
	Score(C)	Positive	42(20.4%)	148(71.8%)	190(92.2%) 16(7.8%)	0.046
		Negative	0(0%)	16(7.8%)		
SP84	Score(A)	Positive	25(12.1%)	87(42.2%)	112(54.3%) 94(45.7%)	0.491
		Negative	17(8.3%)	77(37.4%)		
	Score(B)	Positive	37(18%)	123(59.7%)	160(77.7%) 46(22.3%)	0.095
		Negative	5(2.4%)	41(19.9%)		
	Score(C)	Positive	42(20.4%)	139(67.5%)	181(87.9%) 25(12.1%)	0.003
		Negative	0(0%)	25(12.1%)		
		Total	42(20.4%)	164(79.6%)		

## Discussion

Personalized therapies are based upon the exact determination, characterization and quantification of the according target molecules. The significance of the assessment of EGFR gene copy number and EGFR protein expression as biomarkers to predict therapy responders as well as the selection of patients who might potentially benefit from EGFR targeted therapies was demonstrated by different studies [21,26–28] and also the FLEX study [9,17]. Additionally, the predicitive value of EGFR as biomarker was previously shown [29]. As a methodological base to identify lung cancer patients who might benefit from EGFR-specific antibodies, we studied the relationship between EGFR-expression on protein level and gene copy numbers assessed by FISH.

As part of a broad investigation, we analyzed EGFR expression in tumor samples of NSCLC patients by IHC using four different EGFR specific antibody clones and three scoring methods and correlated these data with FISH analysis. The comprehensive comparison of Dako PharmDx, 31G7, 2.1E1 and SP84 analyzed by different scoring methods has not previously been published just as matching two different FISH probes. Dako PharmDx and 31G7 are frequently used antibodies for EGFR-expression studies [6,9]. 2.1E1 and SP84 are both commercially available and therefore they are also candidates to be applied in the characterization of clinical routine paraffine sections to evaluate EGFR-expression.

The evaluation of the four antibody clones demonstrated the highest degree of agreement between Dako PharmDx and 31G7 amounting to 83.5% applying scoring method (A), to 94.2% with score (B), and to 98.5% with score (C) which was previously also shown by Lee et al. [18]. Both antibodies show similar sensitivity in EGFR staining in formalin fixed NSCLC specimens. 2.1E1 showed the highest sensitivity when scoring methods (A) (85.9%) and (C) (92.9%) were used. Dako PharmDx and 31G7 have nearly the same EGFR staining intensity as previously described by Lee et al. [18], even after evaluation with the three scoring methods.

SP84 showed weaker binding to the EGFR, independent of the scoring method. That might be due to the fact, that SP84 is generated against a synthetic peptide corresponding to C-terminus of human EGFR protein, whereas 2.1E1, Dako PharmDx and 31G7 recognize the extracellular domain of the molecule.

Our results demonstrated significant correlations for EGFR staining results between the four antibodies (p < 0.001). These data are in compliance with Hirsch et al. [6]. In this study, Dako PharmDx and 31G7 and two different scoring systems were compared. They postulated that lower cut off points for Dako PharmDx provide the best discrimination between EGFR positive and negative patients and therefore generate more accurate prediction of survival dependent on gefitinib treatment.

In accordance with other studies [19,21,30], we showed a more frequent positive EGFR expression for all of the four antibodies in SCC than in ADC when scoring method (A) was executed. Just for 2.1E1, scored with method A, positive EGFR expression was equal distributed between SCC and ADC. Whereas evaluation with scoring method (C) indicated a much more prominent EGFR expression in ADC than in SCC. Scoring with method (B) showed a similar EGFR expression in SCC and ADC for Dako PharmDx and 31G7. 2.1E1 features a higher EGFR expression in ADC than in SCC, whereas SP84 indicates an EGFR overexpression more frequently in SCC than in ADC. Although, there were no significant differences in EGFR expression between the ADC subtypes according to IASLC classification [31], EGFR overexpression was most frequently in the acinar subtype, followed by the papilary and solid subtypes. This incidence was consistent for all of the four antibodies and the three scoring methods. Warth et al. had previously shown that the novel histologic IASLC/ATS/ERS classification of pulmonary ADC has prognostic impact [32]. The significance of EGFR expression on protein level as prognostic and also as predictive marker in NSCLC is emphasized by Travis et al. [12] and necessitates further studies to obtain more insights in the correlation of EGFR expression within ADC subtypes.

According to the Colorado scoring system of Varella-Garcia et al. [24] and Cappuzzo et al. [22], both EGFR specific FISH probes showed exactly the same results. The consistency of both FISH probes according to the results of allocation into the same subtypes of disomy, low and high trisomy, low and high polysomy and amplification is disambiguate. To our knowledge, this is the first study that compared the results of two different FISH probes. These data underline that FISH analysis is a reliable and reproducible technique to evaluate EGFR gene amplification in comparison to the determination of EGFR status by IHC.

In this study, 42 tumor samples (20.4%) were FISH positive as represented by high polysomy (13.6%), low amplification (42.4%) and high amplification (4.3%). These results are in concordance with Lee et al. [18] and Hirsch et al. [21]. In our study, EGFR positivity was more frequent in ADC than in SCC (11.2% vs. 8.3%) which is also in accordance with Lee et al. [18] but is contrary to the results of Hirsch et al. [21] in which EGFR amplification is more frequent in SCC.

We were able to show significant differences concerning EGFR FISH patterns in adenocarcinoma subtypes (p = 0.029). These results differ from the study of Soma et al. [33] in which EGFR gene amplification did not differ among predominant patterns. Our results demonstrated an explicit relationship between EGFR gene copy number and ADC subtypes, thus warrant further investigations.

Comparison of IHC data of Dako PharmDx, 31G7 and 2.1E1 and the results obtained by FISH analysis showed strong association between both methods. 31G7 showed the highest correlation to FISH analysis when evaluated with score B (p = 0.001). Whereas IHC data performed with SP84 showed significant correlation only after scoring with method C. Again, this antibody clone is out of band and the reason therefore might be the different epitope which is recognized by SP84.

FISH analysis in combination with IHC independent of the choice of antibody Dako PharmDx, 31G7 and 2.1E1, and regardless of which scoring method, currently seems to be the best approach to identify patients that might profit of EGFR target therapies. The standardization of EGFR status determination is compulsory. Our advice is to use Dako PharmDx, 31G7 or 2.1E1 in IHC and confirm these data by FISH analysis to facilitate more patients for EGFR specific treatments concerning their EGFR expression pattern. However these findings have to be correlated with clinical outcomes following treatment with EGFR-antibodies in order to validate the predictive quality of the EGFR expression status.

## Conclusions

In summary, the message of this study is: different methods exist for the evaluation of EGFR expression leading to different results. Highest concordance with FISH is shown for antibody clone 31G7, evaluated with score B (p = 0.001). Thus, IHC performed with this antibody might be used as standard for the determination of EGFR expression. Additionally, we showed, that there is no correlation between EGFR expression and histological subtypes and clinicopathological data. For future treatment-studies investigating the efficacy of EGFR specific antibodies, the choice of a standardized antibody for EGFR-IHC is therefore crucial to ensure the comparability of EGFR-expression results.

In a next step we will compare formalin fixed paraffin embedded versus HOPE fixed lung tissue with intention to identify differences between the four investigated antibodies between these two different fixation methods. Further investigations will focus on the evaluation of EGFR expression on mRNA-level by real time PCR and western blot analysis to validate these data. Additionally, mutation analysis of compound EGFR mutations [34] and EGFR downstream genes [35] will be performed in order to predict response rates to TKI therapies. Previous studies figured out, that phosphorylation of EGFR is also associated with poor outcome in NSCLC [36]. Surprisingly, 10%-20% of NSCLC patients with EGFR wild type NSCLC also benefit from TKIs [37,38]. Therefore, it is crucial to combine all these EGFR characterization methods to get more insight into the correlation of EGFR expression on protein level, gene amplification, activation status and response rates to EGFR selective therapeutics. For this purpose, Short Term Stimulation of Tissue (STST) could be performed. All these comprehensive investigations have high relevance to improve the identification of more patients who might profit of EGFR specific therapies in future.

## Abbreviations

EGFR: Epidermal Growth Factor Receptor
IHC: Immunohistochemistry
FISH: Fluorescence In Situ Hybridization
NSCLC: Non-small cell lung cancer
TKI: Tyrosine kinase inhibitor
FFPE: Formalin-fixed, paraffin-embedded
TMA: Tissue microarray
ADC: Adenocarcinoma
SCC: Squamous cell carcinoma
LCC: Large cell carcinoma
DAPI: 4′,6-diamidino-2-phenylindole
FLEX: First Line Treatment for Patients with EGFR-expressing Advanced NSCLC

## References

[1] Herbst RS, Heymach JV, Lippman SM (2008). Lung cancer. N Engl J Med.

[2] Schiller JH, Harrington D, Belani CP, Langer C, Sandler A, Krook J, Zhu J, Johnson DH, Eastern Cooperative Oncology Group (2002). Comparison of four chemotherapy regimens for advanced non-small-cell lung cancer. N Engl J Med.

[3] Wells A (1999). EGF receptor. Int J Biochem Cell Biol.

[4] Nicholson RI, Gee JM, Harper ME (2001). EGFR and cancer prognosis. Eur J Cancer.

[5] Hynes NE, Lane HA (2005). ERBB receptors and cancer: the complexity of targeted inhibitors. Nat Rev Cancer.

[6] Hirsch FR, Dziadziuszko R, Thatcher N, Mann H, Watkins C, Parums DV, Speake G, Holloway B, Bunn PA, Franklin WA (2008). Epidermal growth factor receptor immunohistochemistry: comparison of antibodies and cutoff points to predict benefit from gefitinib in a phase 3 placebo-controlled study in advanced nonsmall-cell lung cancer. Cancer.

[7] Mendelsohn J (2002). Targeting the epidermal growth factor receptor for cancer therapy. J Clin Oncol.

[8] Baselga J, Arteaga CL (2005). Critical update and emerging trends in epidermal growth factor receptor targeting in cancer. J Clin Oncol.

[9] Pirker R, Pereira JR, von Pawel J, Krzakowski M, Ramlau R, Park K, de Marinis F, Eberhardt WE, Paz-Ares L, Storkel S, Schumacher KM, von Heydebreck A, Celik I, O’Byrne KJ (2012). EGFR expression as a predictor of survival for first-line chemotherapy plus cetuximab in patients with advanced non-small-cell lung cancer: analysis of data from the phase 3 FLEX study. Lancet Oncol.

[10] Kersting C, Packeisen J, Leidinger B, Brandt B, von Wasielewski R, Winkelmann W, van Diest PJ, Gosheger G, Buerger H (2006). Pitfalls in immunohistochemical assessment of EGFR expression in soft tissue sarcomas. J Clin Pathol.

[11] Meert AP, Martin B, Delmotte P, Berghmans T, Lafitte JJ, Mascaux C, Paesmans M, Steels E, Verdebout JM, Sculier JP (2002). The role of EGF-R expression on patient survival in lung cancer: a systematic review with meta-analysis. Eur Respir J.

[12] Travis WD, Brambilla E, Noguchi M, Nicholson AG, Geisinger K, Yatabe Y, Ishikawa Y, Wistuba I, Flieder DB, Franklin W, Gazdar A, Hasleton PS, Henderson DW, Kerr KM, Petersen I, Roggli V, Thunnissen E, Tsao M (2013). Diagnosis of lung cancer in small biopsies and cytology: implications of the 2011 International Association for the Study of Lung Cancer/American Thoracic Society/European Respiratory Society classification. Arch Pathol Lab Med.

[13] Mirsadraee S, Oswal D, Alizadeh Y, Caulo A, van Beek E (2012). The 7th lung cancer TNM classification and staging system: Review of the changes and implications. World J Radiol.

[14] Hofman P, Butori C, Havet K, Hofman V, Selva E, Guevara N, Santini J, Van Obberghen-Schilling E (2008). Prognostic significance of cortactin levels in head and neck squamous cell carcinoma: comparison with epidermal growth factor receptor status. Br J Cancer.

[15] Modjtahedi H, Khelwatty SA, Kirk RS, Seddon AM, Essapen S, Del Vecchio CA, Wong AJ, Eccles S (2012). Immunohistochemical discrimination of wild-type EGFR from EGFRvIII in fixed tumour specimens using anti-EGFR mAbs ICR9 and ICR10. Br J Cancer.

[16] Steffensen KD, Waldstrom M, Olsen DA, Corydon T, Lorentzen KA, Knudsen HJ, Jeppesen U, Brandslund I, Jakobsen A (2008). Mutant epidermal growth factor receptor in benign, borderline, and malignant ovarian tumors. Clin Cancer Res.

[17] Pirker R, Pereira JR, Szczesna A, von Pawel J, Krzakowski M, Ramlau R, Vynnychenko I, Park K, Yu CT, Ganul V, Roh JK, Bajetta E, O’Byrne K, de Marinis F, Eberhardt W, Goddemeier T, Emig M, Gatzemeier U (2009). Cetuximab plus chemotherapy in patients with advanced non-small-cell lung cancer (FLEX): an open-label randomised phase III trial. Lancet.

[18] Lee HJ, Xu X, Choe G, Chung DH, Seo JW, Lee JH, Lee CT, Jheon S, Sung SW, Chung JH (2010). Protein overexpression and gene amplification of epidermal growth factor receptor in nonsmall cell lung carcinomas: Comparison of four commercially available antibodies by immunohistochemistry and fluorescence in situ hybridization study. Lung Cancer.

[19] Dacic S, Flanagan M, Cieply K, Ramalingam S, Luketich J, Belani C, Yousem SA (2006). Significance of EGFR protein expression and gene amplification in non-small cell lung carcinoma. Am J Clin Pathol.

[20] Li AR, Chitale D, Riely GJ, Pao W, Miller VA, Zakowski MF, Rusch V, Kris MG, Ladanyi M (2008). EGFR mutations in lung adenocarcinomas: clinical testing experience and relationship to EGFR gene copy number and immunohistochemical expression. J Mol Diagn.

[21] Hirsch FR, Chitale D, Riely GJ, Pao W, Miller VA, Zakowski MF, Rusch V, Kris MG, Ladanyi M (2003). Epidermal growth factor receptor in non-small-cell lung carcinomas: correlation between gene copy number and protein expression and impact on prognosis. J Clin Oncol.

[22] Cappuzzo F, Hirsch FR, Rossi E, Bartolini S, Ceresoli GL, Bemis L, Haney J, Witta S, Danenberg K, Domenichini I, Ludovini V, Magrini E, Gregorc V, Doglioni C, Sidoni A, Tonato M, Franklin WA, Crino L, Bunn PA, Varella-Garcia M (2005). Epidermal growth factor receptor gene and protein and gefitinib sensitivity in non-small-cell lung cancer. J Natl Cancer Inst.

[23] Varella-Garcia M (2006). Stratification of non-small cell lung cancer patients for therapy with epidermal growth factor receptor inhibitors: the EGFR fluorescence in situ hybridization assay. Diagn Pathol.

[24] Varella-Garcia M, Diebold J, Eberhard DA, Geenen K, Hirschmann A, Kockx M, Nagelmeier I, Ruschoff J, Schmitt M, Arbogast S, Cappuzzo F (2009). EGFR fluorescence in situ hybridisation assay: guidelines for application to non-small-cell lung cancer. J Clin Pathol.

[25] Drum ML, Shiovitz-Ezra S, Gaumer E, Lindau ST (2009). Assessment of smoking behaviors and alcohol use in the national social life, health, and aging project. J Gerontol B Psychol Sci Soc Sci.

[26] Hirsch FR, Varella-Garcia M, Cappuzzo F, McCoy J, Bemis L, Xavier AC, Dziadziuszko R, Gumerlock P, Chansky K, West H, Gazdar AF, Crino L, Gandara DR, Franklin WA, Bunn PA (2007). Combination of EGFR gene copy number and protein expression predicts outcome for advanced non-small-cell lung cancer patients treated with gefitinib. Annals Oncol.

[27] Barber TD, Vogelstein B, Kinzler KW, Velculescu VE (2004). Somatic mutations of EGFR in colorectal cancers and glioblastomas. N Engl J Med.

[28] Tsuchihashi Z, Khambata-Ford S, Hanna N, Janne PA (2005). Responsiveness to cetuximab without mutations in EGFR. N Engl J Med.

[29] Warth A, Penzel R, Lindenmaier H, Brandt R, Stenzinger A, Herpel E, Goeppert B, Thomas M, Herth FJ, Dienemann H, Schnabel PA, Schirmacher P, Hoffmann H, Muley T, Weichert W (2014). EGFR, KRAS, BRAF and ALK gene alterations in lung adenocarcinomas: patient outcome, interplay with morphology and immunophenotype. Eur Respir J.

[30] Jeon YK, Sung SW, Chung JH, Park WS, Seo JW, Kim CW, Chung DH (2006). Clinicopathologic features and prognostic implications of epidermal growth factor receptor (EGFR) gene copy number and protein expression in non-small cell lung cancer. Lung Cancer.

[31] Lee HJ, Lee CH, Jeong YJ, Chung DH, Goo JM, Park CM, Austin JH (2012). IASLC/ATS/ERS International Multidisciplinary Classification of Lung Adenocarcinoma: novel concepts and radiologic implications. J Thorac Imaging.

[32] Warth A, Muley T, Meister M, Stenzinger A, Thomas M, Schirmacher P, Schnabel PA, Budczies J, Hoffmann H, Weichert W (2012). The novel histologic International Association for the Study of Lung Cancer/American Thoracic Society/European Respiratory Society classification system of lung adenocarcinoma is a stage-independent predictor of survival. J Clin Oncol.

[33] Soma S, Tsuta K, Takano T, Hatanaka Y, Yoshida A, Suzuki K, Asamura H, Tsuda H (2014). Intratumoral distribution of EGFR-amplified and EGFR-mutated cells in pulmonary adenocarcinoma. Pathol Res Pract.

[34] Kobayashi S, Canepa HM, Bailey AS, Nakayama S, Yamaguchi N, Goldstein MA, Huberman MS, Costa DB (2013). Compound EGFR mutations and response to EGFR tyrosine kinase inhibitors. J Thorac Oncol.

[35] Kim HR, Cho BC, Shim HS, Lim SM, Kim SK, Chang J, Kim DJ, Kim JH (2014). Prediction for response duration to epidermal growth factor receptor-tyrosine kinase inhibitors in EGFR mutated never smoker lung adenocarcinoma. Lung Cancer.

[36] Kanematsu T, Yano S, Uehara H, Bando Y, Sone S (2003). Phosphorylation, but not overexpression, of epidermal growth factor receptor is associated with poor prognosis of non-small cell lung cancer patients. Oncol Res.

[37] Holbro T, Hynes NE (2004). ErbB receptors: directing key signaling networks throughout life. Annu Rev Pharmacol Toxicol.

[38] Sequist LV, Bell DW, Lynch TJ, Haber DA (2007). Molecular predictors of response to epidermal growth factor receptor antagonists in non-small-cell lung cancer. J Clin Oncol.

